# Preexisting Liver Disease and Pregnancy: Optimizing Care to Optimize Outcomes

**DOI:** 10.5152/tjg.2026.25753

**Published:** 2026-01-02

**Authors:** Ilkay Ergenc, Alexandra Frolkis, Michael Heneghan

**Affiliations:** 1 The Roger Williams Institute of Liver Studies, King’s College Hospital, London, UK; 2 Division of Gastroenterology and Hepatology, Department of Medicine, University of Calgary, Calgary, Canada; 3 School of Immunology and Microbial Sciences, King’s College Faculty of Life Sciences and Medicine, London, UK

**Keywords:** Chronic liver disease, cirrhosis, drug safety, fetal outcomes, hepatic function, liver transplantation, multidisciplinary care, perinatal monitoring, portal hypertension, pregnancy

## Abstract

Preexisting liver disease, particularly the presence of cirrhosis and portal hypertension (PHT), presents a significant challenge during gestation, necessitating close collaboration between obstetric and hepatology/gastroenterology teams. Women with underlying liver conditions face an increased likelihood of adverse maternal and fetal outcomes. Proactive identification and management of the potential risks associated with pregnancy in this patient group is therefore crucial. While the desire to start a family is deeply personal, each pregnancy carries unique risks requiring careful consideration. Although no single recommendation can comprehensively address all forms of liver disease, pregnancy in women with underlying hepatic conditions can be both safe and achievable when managed through a multidisciplinary team approach, involving the mother and her partner from the preconception stage through to the postpartum period. This review summarizes the current evidence regarding the risks and management strategies for pregnancy complicated by preexisting liver disease, including cirrhosis and PHT, steatotic liver disease, viral hepatitis, autoimmune liver disease, and liver transplant recipients.

**Main Points**

- Women with preexisting liver disease face an increased relative risk of adverse maternal and fetal outcomes.
- Preconception counseling is strongly recommended for women of reproductive age to ensure optimal disease control and to adjust treatment plans for compatibility with pregnancy and make appropriate delivery planning.
- Physiological and biochemical adaptations during pregnancy alter liver function parameters and enzyme profiles, complicating the interpretation of laboratory values and the recognition of higher-risk conditions.
- The presence of uncontrolled maternal disease significantly compromises maternal and fetal outcomes; therefore, therapeutic strategies during pregnancy must balance the potential risk to the fetus with the clinical imperative of effective disease control.
- Optimal pregnancy timing and effective management of liver disease across the preconception, antenatal, and postnatal periods, coordinated by a multidisciplinary team, are essential for achieving favorable maternal and fetal outcomes.
- A disease- and trimester-specific framework for assessment enables clinicians to establish an individualized treatment and monitoring plan for women with preexisting liver disease.

## Introduction

The connection between liver health and fertility has been recognized since antiquity. Indeed, in Babylonian, Assyrian, and Ancient Greek traditions, the liver’s condition and color in hepatoscopy, the examination of sacrificed sheep’s livers as a horoscopy, were considered prophetic of both barrenness and fertility.^1^

Pregnancy induces extensive and dynamic physiological changes affecting nearly every maternal organ system. These adaptations are essential for supporting fetal growth, maintaining maternal homeostasis, and preparing the body for parturition. The liver occupies a central, integrative role in maternal physiology, being fundamental to metabolic, endocrine, and immunological regulation.

In women with preexisting liver disease, particularly advanced chronic liver disease (ACLD) with portal hypertension (PHT), hepatic functional reserve is often limited.^2^ The additional hemodynamic, metabolic, and immunological demands imposed by pregnancy may exceed this reserve, potentially precipitating severe maternal and fetal complications.

**Figure 1.**
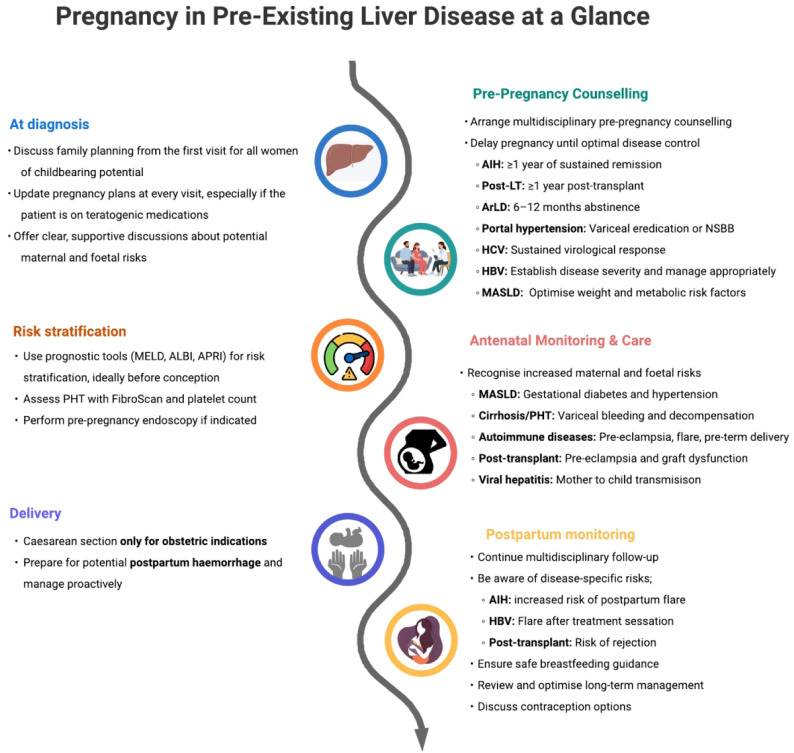
Pregnancy in liver disease at a glance. This figure summarizes the key elements of managing pregnancy in women with preexisting liver disease, from preconception planning and risk stratification through antenatal monitoring to the postpartum period. AIH, autoimmune hepatitis; ALBI, albumin–bilirubin score; APRI, aspartate aminotransferase to platelet ratio index; ArLD, alcohol-related liver disease; HBV, hepatitis B virus; HCV, hepatitis C virus; LT, liver transplant; MASLD, metabolic dysfunction-associated steatotic liver disease; MELD, Model for End-Stage Liver Disease; PHT, portal hypertension.

This review provides an overview of the hormonal, circulatory, and immunological adaptations that occur during pregnancy, examines the impact of preexisting liver disease on gestation, and discusses strategies to optimize both maternal and fetal outcomes (Figure 1).

### Physiological Changes in Pregnancy

During pregnancy, profound hormonal, immunological, and homeostatic adaptations occur to support fetal development and prepare the mother for childbirth.

Hormonal changes include marked elevations in estrogen, progesterone, and prolactin throughout pregnancy and human chorionic gonadotropin peaks in the first trimester.^3^ Elevated estrogen and progesterone cause systemic vasodilation, which reduces peripheral vascular resistance. This is accompanied by compensatory increases in blood volume and cardiac output. Hyperestrogenism also affects hepatic synthetic and excretory functions, which may manifest clinically as palmar erythema and spider nevi.

Pregnancy induces a state of maternal immune adaptation, facilitating maternal immunological tolerance of the semi-allogeneic fetus. This is primarily achieved through a shift from a Th1-dominant pro-inflammatory response toward a Th2-dominant anti-inflammatory state, accompanied by corresponding changes in the cytokine milieu.^4^ Adaptive immune responses are relatively downregulated, while innate immune cells, including neutrophils and monocytes, exhibit enhanced activity and redistribution, maintaining effective host defense without compromising fetal tolerance.^5^^,^^6^

Hemostasis in pregnancy is characterized by a hypercoagulable state, with increases in fibrinogen and procoagulant clotting factors, accompanied by reductions in natural anticoagulants, including protein C, protein S, antithrombin III, and fibrinolytic activity.^7^

Liver function tests and liver enzymes are also influenced by the physiological changes of pregnancy.^8^ Prothrombin time and international normalized ratio generally remain stable. Serum bilirubin levels may remain within the normal range or show a slight decrease throughout gestation, while serum albumin gradually declines across all trimesters. Aminotransferases, including aspartate aminotransferase (AST) and alanine aminotransferase, typically remain within the normal range; however, the upper limit of normal is reduced by approximately 25% during pregnancy. Gamma-glutamyl transferase also remains stable or may decrease slightly. Alkaline phosphatase increases 2- to 4-fold, due to placental production rather than hepatic dysfunction. Alpha-fetoprotein is not a reliable marker in pregnancy, as its levels increase physiologically due to fetal production. Platelet counts typically remain stable; however, pregnancy is associated with a reduction in hemoglobin and an increase in white blood cell counts (Table 1).^2^^,^^9^Pregnancy is associated with a physiological hyperlipidemia, driven largely by hormonal changes that support fetal growth and development. Total cholesterol levels typically rise progressively throughout gestation, particularly in the second and third trimesters, often reaching 30%-50% above pre-pregnancy values.

Increased hormonal levels during pregnancy affect the hepatobiliary system at both cellular and ductular levels. Impaired function of bile salt export pumps may lead to a mild increase in serum bile acids. In addition, smooth muscle relaxation induced by pregnancy hormones reduces gallbladder contractility, increasing the susceptibility to bile stasis and gallstone formation.^10^

### Diagnostic Work-up During Pregnancy

Evaluation of liver disease in pregnancy requires careful selection of diagnostic tests, balancing maternal and fetal safety. Laboratory tests remain the first-line assessment; however, interpretation of results should consider the physiological changes associated with pregnancy (Table 1). ^2^^,^^9^

Imaging studies are generally safe when non-ionizing modalities are used. Doppler ultrasonography is the preferred first-line imaging technique for assessing hepatic structure, the biliary system, and vascular anatomy. Magnetic resonance imaging (MRI) without gadolinium can be considered when further evaluation is required. The MRI during the first trimester is generally avoided unless clinically essential, due to a theoretical risk of fetal warming, although no evidence has shown an increased risk of fetal or early childhood harm.^11^ Radiation exposure from computed tomography (CT) is typically between 10 mGy and 50 mGy, which is well below the teratogenic threshold (>200 mGy). Based on observational data and animal studies, doses exceeding 100 mGy, and particularly those above 150 mGy, are regarded as the minimal levels at which adverse fetal effects may begin to occur.^12^ Consequently, CT can be performed when the potential diagnostic benefit outweighs the associated risk. Ideally, it should be reserved for cases in which MRI is inconclusive or technically not feasible. Endoscopic retrograde cholangiopancreatography can be performed safely during pregnancy when there is a clear clinical indication. It should be carried out with appropriate maternal positioning and minimal sedation, preferably during the second or third trimester.^13^^,^^14^ There are insufficient data regarding the safety of gadolinium or iodinated contrast agents, and their use during pregnancy is currently not recommended.^15^

FibroScan® (transient elastography) can be used to assess liver stiffness during pregnancy and is considered safe; however, results should be interpreted with caution due to the lack of established reference ranges in this population.^16^

Invasive procedures should be reserved for essential indications. Liver biopsy is not absolutely contraindicated in pregnancy but should be deferred until after delivery if possible. A large European cohort reported an increased risk of preterm birth and small for gestational age infants, although the contribution of the underlying liver disease to these outcomes remains uncertain.^17^ When clinically required, biopsy is preferably performed in the second trimester to minimize procedural risk.

Safety data on paracentesis during pregnancy are limited; therefore, pregnancy is generally considered a relative contraindication.^18^ Evidence from cases of ovarian hyperstimulation syndrome suggests that paracentesis increases uterine perfusion and does not adversely affect pregnancy outcomes.^19^ Consequently, paracentesis can be performed during pregnancy when the potential benefits outweigh the risks by experienced clinicians, with standard precautions in place.

Esophagogastroduodenoscopy and flexible sigmoidoscopy are considered safe during pregnancy when clinically indicated. The procedure should be undertaken with careful left lateral decubitus positioning and minimal sedation. Midazolam is the most used sedative agent and is generally well tolerated without significant adverse effects, while pethidine and propofol are acceptable alternatives.

A multidisciplinary approach involving hepatology, obstetrics, and anesthesia is recommended to ensure safe and effective diagnostic evaluation during pregnancy. ^9^^,^^14^^,^^20^

### General Principles of Managing Preexisting Liver Disease in Pregnancy

A comprehensive preconception assessment is fundamental in women with preexisting liver disease. Ideally, preconception counseling should be conducted jointly by hepatology and obstetric teams.^21^ This multidisciplinary approach ensures accurate risk assessment, safe treatment planning, and clear communication regarding anticipated pregnancy outcomes. Medication should be carefully reviewed for potential teratogenicity risk and adjusted prior to conception (Table 2).

The primary aim is to achieve stable disease control and minimize both the fetal and maternal risks before conception. Pregnancy should ideally be planned at least a year after sustained remission. Active and/or uncontrolled disease at conception is associated with an increased risk of maternal decompensation, miscarriage, preterm delivery, and low birth weight. Where possible, treatment regimens should be reviewed and adjusted to the lowest effective doses of agents with established safety profiles in pregnancy. Drugs with potential teratogenicity should be discontinued or substituted with safer alternatives in advance. Women planning a family should be assessed for immunity to hepatitis A, hepatitis B, and other vaccine-preventable infections. Non-live vaccines may be administered safely prior to or during pregnancy if indicated, whereas live vaccines should be given at least 1 month before conception.

In women with ACLD, variceal surveillance should be performed within 1 year prior to conception, with appropriate management of any identified varices. This may include non-selective beta-blocker therapy or endoscopic variceal ligation (EVL), depending on individual tolerance and clinical context. In addition, cross-sectional imaging should be undertaken to evaluate the extent of PHT and to assess for the presence of collateral circulation, particularly in anatomical regions relevant to cesarean section planning.

### Steatotic Liver Diseases

#### Metabolic Dysfunction–Associated Steatotic Liver Disease:

Metabolic dysfunction-associated steatotic liver disease (MASLD) has emerged as the most prevalent liver disease globally. It represents a growing public health concern, with both its prevalence and incidence rising rapidly across all populations, including women of reproductive age. The prevalence of MASLD in pregnancy nearly tripled between 2007 and 2016,^22^ and recent studies have reported rates approaching 30%.^23^ The MASLD is strongly associated with obesity, type 2 diabetes mellitus (DM), and metabolic syndrome, with particularly high rates observed among individuals with obesity. In England, approximately one-quarter of women are already living with obesity at the beginning of pregnancy.^24^

Metabolic dysfunction-associated steatotic liver disease encompasses a wide histopathological spectrum, ranging from simple steatosis to steatohepatitis and ACLD. Beyond hepatic involvement, the accompanying metabolic dysfunction, including insulin resistance, hypertension, and overweight or obesity, plays a central role in the development of adverse maternal and fetal outcomes.

Metabolic dysfunction-associated steatotic liver disease in pregnancy has been linked with an increased risk of gestational DM (GDM), gestational hypertension, pre-eclampsia, cesarean section, postpartum hemorrhage, large for gestational age (LGA), and preterm birth. A meta-analysis of 22 studies demonstrated that MASLD was associated with more than a 3-fold increase in the risk of GDM and pre-eclampsia, and a 2-fold increase in the risk of premature birth and LGA infants.^25^ A nationwide Swedish study further confirmed MASLD as an independent risk factor for preterm birth, irrespective of obesity or other metabolic comorbidities.^26^

Women with MASLD should be carefully evaluated for steatohepatitis, hepatic fibrosis, or ACLD, as well as for coexisting metabolic abnormalities. Lifestyle modification remains the cornerstone of management and should be strongly encouraged to mitigate both hepatic and systemic risk factors. Recently, resmetirom has been approved for adults with non-cirrhotic metabolic dysfunction-associated steatohepatitis (MASH) and fibrosis stage ≥2; however, lifestyle intervention remains the most effective strategy for improving hepatic and cardiometabolic outcomes.^27^ In patients with obesity, Glucagon-Like Peptide-1 (GLP-1) receptor agonists may support weight loss and improve associated metabolic risk factors, including MASLD. The European Endocrine guidelines recommend stopping GLP-1 agonists 2 months before conception.^28^ A weight reduction of ≥5% is recommended to improve hepatic steatosis, while ≥7%-10% is advised to achieve histological improvement in MASH and fibrosis.^29^

During pregnancy, no pharmacological therapies have yet been approved or proven safe for MASLD or obesity. Management should therefore focus on optimizing metabolic health through diet and physical activity, while avoiding weight loss interventions that could compromise fetal growth. European guidelines recommend pregnant women with MASLD incorporate the same lifestyle interventions into their routines as non-pregnant women. Both European and American guidelines advise against consuming processed foods, artificially sweetened beverages, refined carbohydrates, excessive saturated fats, and fructose,^81^ which are associated with a higher risk of MASLD.^30^^,^^31^

Pregnancy in women with MASLD requires assessment for GDM and hypertension. Breastfeeding should be encouraged, as lactation for at least 6 months appears to confer a protective effect against the subsequent development of MASLD in mothers and has protective effects on offspring MASLD development.^21^^,^^29^

#### Alcohol–related Liver Disease:

Excessive alcohol consumption causes a wide range of liver damage, including steatosis, alcohol-associated hepatitis, and cirrhosis. Alcohol use among women of reproductive age has become an increasing public health concern. Data from national surveys in the United States indicate that between 8.4% and 14% of women report alcohol use during pregnancy, with approximately 4.8% engaging in binge drinking.^32^

Alcohol consumption is associated with reduced fertility, and the presence of alcohol-related liver disease (ArLD) further diminishes conception rates. Alcohol is a potent teratogen known to cause neurodevelopmental disorders and congenital malformations. There is no established safe threshold for alcohol consumption during pregnancy.^33^ Even in the absence of established liver disease, alcohol use during pregnancy carries significant maternal and fetal risks. Maternal complications include an increased risk of GDM, cesarean delivery, antepartum hemorrhage, preterm birth, and stillbirth. Fetal adverse outcomes comprise higher rates of miscarriage, preterm delivery, intrauterine growth restriction, low birth weight, SGA infants, and poor Apgar scores.^34^ Long-term consequences include cognitive, behavioral, and social impairments associated with fetal alcohol spectrum disorder (FASD). It is estimated that between 1% and 5% of first-grade children in the United States are affected by FASD.^35^ The presence of ArLD further amplifies these risks, particularly in women with acute alcoholic hepatitis or alcohol-related cirrhosis.

All women should be screened for alcohol use during pregnancy. Those consuming more than 20 g of alcohol per day should be assessed for ArLD, including evaluation of liver enzymes and fibrosis. Preconception counseling and conception after a sustained period of abstinence represent the safest approach. The cornerstone of ArLD management is sustained abstinence. Management of alcohol use disorder (AUD) in pregnancy primarily relies on psychosocial interventions. Nevertheless, pharmacological therapy should not be avoided when clinically necessary and can be considered in selected cases. Disulfiram is contraindicated due to its association with fetal abnormalities. Limited data on acamprosate use in pregnancy have not demonstrated fetal abnormalities.^36^ Evidence from opioid use disorder treatment indicates no increased risk of adverse fetal outcomes with naltrexone use.^37^ Therefore, naltrexone and acamprosate can be considered in carefully selected high-risk pregnant women with AUD, given its favorable risk–benefit profile compared with the known teratogenicity of alcohol. Withdrawal symptoms can be managed with benzodiazepines.^9^

Alcohol-related hepatitis should be managed according to the same principles applied in nonpregnant patients, with careful consideration of maternal and fetal safety.

### Viral Hepatitis

Although this is the era of global viral hepatitis elimination, chronic viral hepatitis remains the second most common cause of chronic liver disease in pregnancy after MASLD. Moreover, mother-to-child transmission (MTCT) continues to represent a major contributor to the worldwide burden of chronic viral hepatitis.^27^

#### Hepatitis B Virus:

Globally, an estimated 254 million people are living with chronic hepatitis B (CHB) infection, with more than one-quarter being women of childbearing age. The prevalence of CHB virus infection, defined by hepatitis B surface antigen (HBsAg) positivity, varies considerably across regions, ranging from around 0.4% in the United Kingdom to more than 11% in some sub-Saharan African countries.^38^

The MTCT remains the principal route of HBV burden worldwide and represents a major risk factor for the persistence of the infection. Therefore, universal screening for HBV is recommended for all pregnant women during the first trimester.^39^

Antiviral therapy (AVT) markedly reduces the risk of transmission.^38^ The risk of MTCT is negligible when maternal HBV DNA levels are below 5.30 log_10_ IU/mL (200 000 IU/mL). The AVT is therefore recommended for women with HBV DNA above this threshold, or for all HBsAg-positive pregnant women in settings where HBV DNA quantification is unavailable. Tenofovir disoproxil fumarate is the antiviral agent of choice during pregnancy.^40^ Emerging data support the efficacy and safety of tenofovir alafenamide in preventing MTCT.^41^Further evidence is warranted regarding fetal outcomes.

Women who meet treatment criteria for CHB virus from a maternal perspective including ongoing necroinflammation, advanced fibrosis, cirrhosis, or increased hepatocellular carcinoma (HCC) risk should commence or continue AVT during pregnancy. In women started on AVT solely to prevent MTCT, treatment may be discontinued after delivery if long-term therapy is not otherwise indicated for CHB.

All infants born to HBV-infected mothers should receive both passive and active immunization with hepatitis B immunoglobulin and complete a vaccine series. Breastfeeding does not represent a major route of transmission and is considered safe for infants who have received appropriate active and passive immunization.^9^ Still, MTCT may occur in mothers with high viral replication or with cracked nipples during breastfeeding.

The benefit of cesarean section in preventing MTCT is limited, with an estimated 23 procedures required to prevent a single case in women with high HBV DNA titers.^42^ Therefore, HBV infection alone is not an indication for cesarean section, and vaginal delivery is not contraindicated.^21^ Measures to prevent MTCT for HBV are uniformly effective in preventing infection by hepatitis D. Breastfeeding should be encouraged in infants born to HBV/HDV-coinfected mothers as well.

Sexual partners should be screened and vaccinated for hepatitis B.

#### Hepatitis C Virus:

Globally, an estimated 50 million people are living with chronic hepatitis C (HCV), which is the second leading viral cause of ACLD and HCC. The HCV is now curable with direct-acting antiviral (DAA) therapy, and there is an ongoing global elimination program aiming for eradication by 2030.^43^ Nevertheless, the burden of HCV remains high in many regions and age groups, including women of childbearing age. The MTCT occurs in approximately 6% of cases,^44^ making vertical transmission an important contributor to new infections, which is estimated at around 74 000 annually.^45^

Only one-third of individuals with HCV are aware of infection;^38^ HCV testing is therefore recommended for all pregnant women as part of standard antenatal care.^21^ The HCV infection during pregnancy is associated with an increased risk of GDM, low birth weight, preterm birth, and neonatal intensive care unit (NICU) admission.^2^ Intrahepatic cholestasis of pregnancy (ICP) is also significantly more common in women with HCV.^46^ Women with known HCV infection prior to conception should be offered AVT to achieve viral clearance, although DAA therapy is not currently recommended during pregnancy.^9^

There is no robust evidence to support the benefit of cesarean section for reducing perinatal transmission.^47^ Cesarean section is therefore not indicated to prevent MTCT. In women coinfected with HCV and HIV, the mode of delivery can be individualized according to maternal HIV and HCV RNA levels. Breastfeeding is considered safe for HCV-infected mothers, including those with HCV/HIV coinfection receiving antiretroviral therapy.^21^

#### Acute Viral Hepatitis in Pregnancy:

Acute viral hepatitis during pregnancy requires careful consideration due to the increased risk of maternal complications, adverse pregnancy outcomes, and potential vertical transmission.

Hepatitis A virus (HAV) generally follows a similar course to that in the non-pregnant population, with acute, self-limiting infection. Acute liver failure (ALF) is very rare, but HAV can increase the risk of prolonged rupture of membranes and preterm labor.^48^ The MTCT of HAV is extremely uncommon, and there is no evidence of transmission via breastfeeding. Cesarean section is not routinely indicated for acute HAV infection unless obstetric reasons exist, and breastfeeding should be encouraged.

Hepatitis E virus (HEV) and herpes simplex virus pose a higher risk of acute hepatitis and progression to ALF during pregnancy compared with non-pregnant individuals. The HEV infection is more common among pregnant women, particularly in highly endemic regions. Infection during pregnancy can lead to ALF, especially when acquired in the third trimester or with HEV genotype 1.^49^ The HEV also increases the risk of premature rupture of membranes, intrauterine growth restriction, preterm delivery, and perinatal mortality. Management is primarily supportive, as ribavirin and interferon alfa are contraindicated in pregnancy. Delivery or therapeutic termination may be considered on an individualized basis to reduce maternal morbidity and mortality. Neither vaginal delivery nor breastfeeding is contraindicated in the context of HEV infection.^9^^,^^21^

### Autoimmune Liver Diseases in Pregnancy

#### Autoimmune Hepatitis:

Autoimmune hepatitis (AIH) is a relatively rare disease, but it predominantly affects women of childbearing age.^50^ The estimated prevalence of AIH during pregnancy ranges between 1.4 and 6.8 per 100 000 pregnancies, and de novo presentations may occur during gestation.^51^

Pregnancy is generally associated with a state of immunotolerance and relative immune downregulation. Consequently, most women with AIH experience a stable and uncomplicated disease course during pregnancy. Nevertheless, disease relapse can occur, particularly in those who discontinue treatment or exhibit poor adherence. The risk of flare is greatest in the early postpartum period (6-12 weeks after delivery), when the rapid fall in progesterone and estrogen levels contributes to a pro-inflammatory rebound.^52^

Women with AIH are at increased risk of gestational complications, including GDM and hypertensive disorders of pregnancy such as preeclampsia and eclampsia. Adverse fetal outcomes including miscarriage, preterm delivery, and low birth weight are more common in women with active or poorly controlled disease.^53^ Loss of biochemical remission in the year preceding conception has been linked to higher rates of adverse pregnancy outcomes and NICU admission.^54^ Risks are further amplified in women with established cirrhosis and PHT. Despite these challenges, with appropriate preconception counseling, disease control, and multidisciplinary management, maternal and fetal outcomes are generally favorable. ^55^

The management of preexisting or first presentation of AIH during pregnancy follows the same principles as in the non-pregnant population, with certain modifications to ensure maternal safety and fetal protection. Corticosteroids and thiopurines remain the cornerstone of therapy.^9^^,^^21^ Predniso(lo)ne should be prescribed at the lowest effective dose required to maintain remission. Azathioprine is considered safe and is not associated with an increased risk of congenital malformations compared with the general population, although a slight increase in ICP has been reported.^2^^,^^56^

Mycophenolate mofetil (MMF) is contraindicated in pregnancy due to a high teratogenic risk, with congenital malformations reported in up to 27% of cases.^57^ A washout period of at least 12 weeks before conception is recommended for women previously treated with MMF. Tacrolimus is regarded as a safe and effective alternative for patients who are intolerant of, or refractory to, azathioprine.^58^

The best pregnancy outcomes are observed in women who achieve and sustain biochemical remission for at least 1 year prior to conception. Low-dose aspirin (150 mg daily), commenced from 11 weeks of gestation, is recommended to reduce the risk of preeclampsia and related hypertensive complications.^2^^,^^21^^,^^31^ Close biochemical monitoring is advised during pregnancy and especially in the first 3 months postpartum to detect asymptomatic flares. No specific interventions are required for infants born to mothers with AIH.

#### Primary Biliary Cholangitis:

Primary biliary cholangitis (PBC) is a rare disease but predominantly affects women (female-to-male ratio 9 : 1), with an estimated global prevalence of 1.9 to 40.2 per 100 000 population.^59^ Up to one-quarter of patients are diagnosed during their reproductive years, and as many as one-third of new diagnoses may be made in the context of pregnancy, reflecting increased medical evaluation during this period.^60^ Pregnancy generally has a benign course in most women with PBC. Over two-thirds experience favorable maternal outcomes, with stable or even improved liver biochemistry and no significant hepatic complications.^61^ However, de novo or worsening pruritus develops in approximately half of affected women. Up to two-thirds experience biochemical or clinical deterioration after delivery. A modestly increased risk of postpartum hemorrhage has also been reported, although this remains uncommon in women with early-stage disease.^62^

From a fetal perspective, PBC is associated with slightly higher rates of preterm delivery and pregnancy loss compared with the general population. Overall prognosis remains favorable and there is no evidence of increased risk of congenital malformations. The principal determinant of maternal and fetal outcomes is the stage and grade of the disease prior to conception. Women with compensated disease and a sustained BR to therapy generally experience uncomplicated pregnancies.^63^

Ursodeoxycholic acid (UDCA) remains the mainstay of therapy for PBC and should be continued throughout pregnancy and breastfeeding. In women with severe or persistent pruritus, management should follow a stepwise approach (Table 3). Optimizing the UDCA dose from 13-15 mg/kg to up to 20 mg/kg daily is the first step, alongside non-pharmacological measures.^9^ Cholestyramine and rifampicin can be introduced safely as second- and third-line options for pruritus. Fibrates may be considered in selected cases during the later stages of pregnancy, where the potential maternal benefit outweighs the theoretical fetal risk, although clinical data remain limited.

Second-line agents such as obeticholic acid, elafibranor, and seladelpar should be avoided during pregnancy due to insufficient human safety data.^64^ Likewise, the use of bezafibrate and fenofibrate should be deferred unless maternal disease is uncontrolled. The PBC may flare after delivery; therefore, postpartum monitoring is essential. Liver biochemistry should be checked regularly, and treatment should be escalated if persistent cholestasis or biochemical relapse occurs. Breastfeeding can be safely continued with UDCA,^65^ but should generally be avoided if second-line agents are introduced, given the lack of safety data in this setting. No specific neonatal precautions are required for infants of mothers with PBC.

#### Primary Sclerosing Cholangitis:

Primary sclerosing cholangitis (PSC) commonly occurs in association with inflammatory bowel disease (IBD). Although PSC predominantly affects men, it can also present in women of childbearing age.

Fertility is not appreciably reduced in women with PSC. Maternal and fetal outcomes are also generally favorable. A Swedish population-based study of 229 births in women with PSC reported a significantly higher risk of preterm delivery (adjusted prevalence odds ratio ~3.6) and an increased rate of cesarean section, but no elevation in congenital malformations, stillbirths, or neonatal mortality.^66^ On the other hand, active IBD before/during pregnancy is associated with miscarriage, stillbirth, preterm birth, and low birth weight/small for gestational age.

Most women with PSC experience stable disease throughout pregnancy, but up to one-third may develop worsening liver-related symptoms, including pruritus and cholangitis. The stage of disease and the presence of active cholangiopathy at conception are key determinants of maternal and fetal outcomes.^67^ Management of PSC in pregnancy should follow the general principles applied to cholestatic liver disease, with specific precautions. The UDCA should be continued throughout pregnancy, as it is safe and may improve biochemical markers and maternal symptoms. Treatment of pruritus should follow the same stepwise approach as in PBC, beginning with UDCA dose optimization and non-pharmacological measures, with cholestyramine or rifampicin considered if required (Table 3). Close monitoring of maternal liver enzymes and bile acid levels is recommended, and obstetric surveillance should be heightened to anticipate potential complications such as preterm delivery.

### Cirrhosis and Portal Hypertension

Advanced chronic liver disease, a broader term, includes both advanced liver fibrosis and clinically established cirrhosis. Compensated ACLD is a state where liver function remains preserved within physiological limits, maintained by adaptive mechanisms. Nevertheless, ACLD carries an intrinsic risk of functional decline and the subsequent development of complications, known as decompensation, driven by the progression of the underlying disease or exhaustion of adaptive capacity. Pregnancy introduces additional physiological adaptations to support fetal and placental development, creating competing demands that further increase the risk of decompensation in women with ACLD. Particularly, hemodynamic and hormonal changes of pregnancy amplify risks associated with PHT.

Cirrhosis frequently causes amenorrhea and reduced fertility; however, recent studies report favorable fertility rates.^68^ Regardless of etiology, women with cirrhosis have substantially higher risks of ICP, pregnancy-induced hypertension, pre-eclampsia, and postpartum hemorrhage. Pregnancies in women with cirrhosis are also associated with increased rates of neonatal mortality, preterm birth, and SGA.^69^^,^^70^

Maternal mortality among pregnant women with cirrhosis has significantly declined over time, and reported 0.89% in a recent meta-analysis.^71^The overall rate of decompensation during pregnancy is around 1.6%; a prior history of decompensation significantly elevates the risk up to 13%-25%.^68^ Pregnancy outcomes correlate closely with the severity of ACLD. Prognostic scoring systems can guide risk assessment and help predict pregnancy outcomes. A pre-pregnancy Model for End-Stage Liver Disease (MELD) score below 6 predicts favorable outcomes, whereas a MELD score above 10 indicates a high likelihood of decompensation during pregnancy.^72^ Similarly, a pre-pregnancy albumin–bilirubin (ALBI) score ≤2.7 predicts an increased chance of live birth, while higher ALBI scores are associated with shorter gestation and preterm delivery.^73^ A pre-pregnancy AST to platelet ratio index (APRI) <0.84 is also a good predictor of term pregnancies.^73^

A combination of liver stiffness measurement <20 kPa and platelet count >150 × 10^9^/L predicts a low likelihood of high-risk varices based on Baveno criteria, although these criteria have not been validated in pregnancy.^74^ Women with a high probability of clinically significant PHT should undergo screening endoscopy within 1 year prior to conception and receive appropriate prophylactic or endoscopic management. In the absence of preconception assessment, screening endoscopy is recommended during the second trimester of pregnancy to identify and manage varices appropriately.^9^Beta-blockers, such as carvedilol or propranolol, can be safely used during pregnancy. In general, carvedilol is preferred due to its lower association with fetal growth restriction.^75^

The MRI can be used to delineate intra-abdominal and pelvic varices and is preferably performed during the second trimester to be aware in case of emergency cesarean section. ACLD itself is not an indication for cesarean section; delivery mode should be guided solely by obstetric indications.^9^^,^^21^ Postpartum hemorrhage occurs in 5%-45% of women with cirrhosis due to multifactorial causes including coagulopathy, ectopic varices, and thrombocytopenia. Management includes blood product transfusion (fresh frozen plasma, platelets), uterotonic agents, and surgical intervention as needed.

Acute variceal bleeding during pregnancy should be managed according to standard treatment protocols, except that terlipressin should be avoided. Immediate resuscitation should be initiated, with prompt administration of octreotide and broad-spectrum antibiotics, and blood transfusion if indicated. The EVL remains the gold-standard endoscopic treatment for acute esophageal variceal bleeding. Cyanoacrylate glue injection can be used for life-threatening gastric variceal bleeding during pregnancy.^9^ In cases of refractory bleeding or failure of endoscopic therapy, transjugular intrahepatic portosystemic shunt may be considered as a rescue intervention, although its use during pregnancy requires careful multidisciplinary evaluation due to potential maternal and fetal risks.

Splenic artery aneurysms (SAAs) are rare but occur more frequently in the setting of PHT. Pregnancy introduces significant hemodynamic and hormonal changes, including elevated cardiac output, augmented splanchnic blood flow, reduced vascular tone, and increased portal pressures secondary to the gravid uterus, that collectively predispose to aneurysm enlargement and rupture, particularly in the third trimester. While rupture is rare, it carries a high maternal mortality rate, reported between 22% and 70%, and is associated with poor fetal outcomes. Current recommendations advocate screening for SAAs in women of childbearing age with PHT.^76^ However, optimal management remains controversial. Although aneurysms exceeding 2 cm are traditionally considered high risk, approximately half of ruptured SAAs measure less than 2 cm, challenging size-based risk stratification. Ideally, intervention should occur prior to conception; nonetheless, emerging consensus supports treatment during pregnancy at any gestational stage, given the substantial maternal–fetal risk associated with rupture.^77^

### Liver Transplant Recipients

In most liver transplant (LT) recipients, fertility and normal menstrual cycles are restored within the first year following transplantation. Nevertheless, pregnancy should be delayed for at least 1 year post-LT to optimize maternal and fetal outcomes. Conception more than 2 years after LT is associated with lower risks of low birth weight, graft rejection, and graft loss, whereas pregnancies occurring within 6 months carry the highest risk of adverse outcomes.^78^

Pregnant LT recipients have an increased risk of gestational hypertension, pre-eclampsia, GDM, ICP, and acute kidney injury.^79^ Among these, pre-eclampsia is the leading cause of preterm delivery. Prophylactic low-dose aspirin (150 mg daily) should be initiated in the first trimester to reduce this risk.^9^

Live birth rates among LT recipients have improved significantly over the past 3 decades, rising from around 60% to over 80%.^80^ Vaginal delivery is generally safe in the post-LT setting. Cesarean section is reserved for standard obstetric indications. Reported cesarean rates range from 20% to 63%.^79^

Rates of rejection during pregnancy and postpartum vary widely, ranging from 0%-20% to 3%-12% respectively.^78^^,^^79^^,^^81^ Rejection episodes are often multifactorial, typically related to reduced immunosuppressive exposure, either from intentional dose reduction or hemodilution during pregnancy. Rejection typically responds well to standard intravenous methylprednisolone regimen or increased immunosuppression.^9^ The MMF is teratogenic and must be stopped at least 12 weeks prior to conception. Other immunosuppressive agents, including azathioprine, cyclosporine, tacrolimus, and prednisolone, should be continued during pregnancy. Women receiving calcineurin inhibitors (cyclosporine or tacrolimus) require close monitoring for hypertension and pre-eclampsia, while those on glucocorticoids should be screened for GDM. Patients taking more than 5 mg prednisolone daily for over 3 weeks are at risk of adrenal suppression, and stress-dose steroids should be considered at the time of delivery.

## Conclusion

The prevalence of pregnancy in women with preexisting chronic liver disease is steadily increasing. Liver diseases pose unique maternal and fetal risks, requiring careful planning, pre-pregnancy counseling and multidisciplinary coordination. Preconception counseling is essential to optimize disease control, adjust medications, and address disease-specific precautions before conception. Risk stratification and management are particularly important in women with cirrhosis and PHT, where the physiological changes of pregnancy may precipitate decompensation and related complications. Cesarean section is not indicated solely based on liver disease and should be reserved for obstetric reasons only. Throughout pregnancy and the postpartum period, close clinical and biochemical monitoring is vital to ensure maternal stability and early detection of complications. Optimal outcomes for both mother and baby rely on a collaborative, multidisciplinary approach involving hepatology, obstetrics, and neonatology teams to deliver individualized, evidence-based care.

## Figures and Tables

**Table 1.** Normal Ranges for Commonly Used Laboratory Tests During Normal Pregnancy

Blood Tests (Normal Range in Non-Pregnant Women)	First Trimester	Second Trimester	Third Trimester
Full blood count			
Hemoglobin (12-15 mg/dL)	11-14, reduction	10.5-14, reduction	10.5-14, reduction
White cell count (4-11 × 10^9^/L)	6-16, increase	6-16, increase	6-16, increase
Platelet count (150-400 × 10^9^/L)	150-140, stable	150-140, stable	150-140, stable
Lymphocyte count (0.7-4.6 × 10^9^/L)	1.1-3.6, reduction	0.9-3.9, reduction	1.1-3.6, reduction
Urea and electrolytes			
Blood urea nitrogen (7-20 mg/dL)	7-12, reduction	3-13, reduction	3-11, reduction
Creatinine (0.5-0.9 mg/dL)	0.4-0.7, reduction	0.4-0.8, reduction	0.4-0.8, reduction
Liver tests			
Bilirubin, total (0.3-1.3 mg/dL)	0.1-0.4, reduction	0.1-0.8, reduction	0.1-1.1, reduction
Albumin (3.5-4.6 g/dL)	2.8-3.7, reduction	2.8-3.7, reduction	2.8-3.7, reduction
AST (7-40 IU/L)	10-28, stable, reduction in upper normal limit	10-29 stable, reduction in upper normal limit	11-30 stable, reduction in upper normal limit
ALT (0-40 IU/L)	6-32, stable, reduction in upper normal limit	6-32, stable, reduction in upper normal limit	6-32, stable, reduction in upper normal limit
GGT (11-50 IU/L)	5-37, stable or reduction	5-43, stable or reduction	3-41, stable or reduction
ALP (30-130 IU/L)	32-100, increase	43-135, increase	133-418, increase
Pro-thrombin time (10-12 seconds)	1-12, stable	1-12, stable	1-12, stable
Bile acids, non-fasting (0-10 μmol/L )	0-19, stable or increase	0-19, stable or increase	0-19, stable or increase
Alpha-fetoprotein (0-44 μg/L )	Increase	Increase	Increase
Inflammatory markers			
C-reactive protein (<10 mg/dL)	Stable	Stable	Stable
Procalcitonin (<0.05, ng/L)	Stable	Stable	Stable
ESR (0-20 mm/hour)	18-48, increase	30-70, increase	30-70, increase

ALP, alkaline phosphatase; ALT, alanine aminotransferase; AST, aspartate aminotransferase; ESR, erythrocyte sedimentation rate; GGT, gamma-glutamyl transferase; INR, international normalized ratio.

**Table 2.** Safety of Commonly Used Medications in Liver Disease During Pregnancy and Breastfeeding

**Medication**	**Pregnancy Safety**	**Breastfeeding Safety**	**Notes**
Predniso(lo)ne	Compatible/each trimester	Compatible	Monitoring of maternal glucose, blood pressure recommended
Budesonide	Compatible/each trimester	Compatible	
Azathioprine	Compatible/each trimester	Compatible	
Mercaptopurine	Compatible/each trimester	Compatible	
Mycophenolate mofetil	Not compatible	Not compatible	
Tacrolimus	Compatible/each trimester	Compatible	Monitoring of maternal glucose, blood pressure, renal function, and drug levels recommended
Cyclosporine	Compatible/each trimester	Compatible	
Sirolimus	Insufficient data/not routinely recommended	Insufficient data	
Infliximab	Compatible	Compatible	Avoid live vaccines in infants for first 6 months
Rituximab	Limited data; use only if benefits outweigh risks	Not recommended	
Everolimus	Insufficient data/not routinely recommended	Insufficient data	
Tenofovir disoproxil fumarate (TDF)	Compatible/each trimester	Compatible	Preferred antiviral for HBV
Tenofovir alafenamide (TAF)	Compatible	Compatible	
Interferon	Limited data/not recommended	Not recommended	
Ribavirin	Not compatible	Not compatible	
Ursodeoxycholic acid	Compatible/each trimester	Compatible	
Bezafibrate	Limited human data; use only if benefits outweigh risks	Not recommended	
Fenofibrate	Limited human data; use only if benefits outweigh risks	Not recommended	
Elafibranor	No human data, not recommended	Not recommended	
Seladelpar	No human data, not recommended	Not recommended	
Colestyramine	Compatible/each trimester	Compatible	Monitoring and treatment of vitamin K deficiency is recommended
Naltrexone	Limited data; use only if benefits outweigh risks	Not recommended	
Rifampicin	Limited data, generally considered safe, should not be withheld if indicated	Compatible	
Aspirin	Compatible/each trimester	Compatible	
Resmetirom	No human data, not recommended	Not recommended	
Trientine *	Limited human data	Limited human data	Anti-copper therapy should be maintained during pregnancy
Penicillamine^ *^	Limited human data	Limited human data	
N-acetylcysteine	Compatible/each trimester	Compatible	
Disulfiram	Not compatible	Not compatible	
Baclofen	Limited human data	Not compatible	
Acamprosate	Limited human data; use only if benefits outweigh risks	Not recommended	

*The benefits of continuing treatment outweigh potential risks. Current evidence does not support advising against breastfeeding in mothers receiving anti-copper therapy.

**Table 3.** Stepwise Management of Cholestatic Pruritus During Pregnancy

1. Non-pharmacological measures	Cold baths/showers Hydrate skin after bathing Use unscented moisturizers Wear loose natural-fibre clothing Maintain lower room temperature Avoid hot/dry environments
2. UDCA dose optimization	Standard dose for PBC/PSC: 13-15 mg/kg/day May increase up to 20 mg/kg/day for pruritus in pregnancy
3. Cholestyramine	4-12 g once daily or divided dose May interfere with absorption of fat-soluble vitamins and UDCA
4. Rifampicin	150-450 mg once daily (max 600 mg/day) Monitor liver function 2-4 weeks after initiation due to hepatotoxicity risk (~10%-15%)
5. Fibrates	Bezafibrate 400 mg once daily Consider in later trimesters if maternal benefits outweigh theoretical fetal risks
6. IBAT inhibitors	Odevixibat and maralixibat are approved IBAT inhibitors Minimally absorbed systemically; however, pregnancy safety data remain limited Should only be considered for severe, refractory cases and only under specialist supervision
7. Nasobiliary drainage	Endoscopic placement of nasobiliary catheter for external bile diversion Should only be considered for intractable pruritus unresponsive to all medical therapies Typically performed in tertiary centers

IBAT, intestinal bile acid transport; PBC, primary biliary cholangitis; PSC, primary sclerosing cholangitis; UDCA, ursodeoxycholic acid.

## Data Availability

The data that support the findings of this study are available on request from the corresponding author.
